# Improved Visibility of Metastatic Disease in the Liver During Intra-Arterial Therapy Using Delayed Arterial Phase Cone-Beam CT

**DOI:** 10.1007/s00270-016-1406-2

**Published:** 2016-07-05

**Authors:** Ruediger E. Schernthaner, Reham R. Haroun, Rafael Duran, Howard Lee, Sonia Sahu, Jae Ho Sohn, Julius Chapiro, Yan Zhao, Boris Gorodetski, Florian Fleckenstein, Susanne Smolka, Alessandro Radaelli, Imramsjah Martijn van der Bom, MingDe Lin, Jean Francois Geschwind

**Affiliations:** 1 Section of Cardiovascular and Interventional Radiology, Department of Biomedical Imaging and Image-guided Therapy, Medical University of Vienna, Währinger Gürtel 18-20, 1090 Vienna, Austria; 2 Department of Radiology and Biomedical Imaging, Yale University School of Medicine, 330 Cedar Street, TE 2-230, New Haven, CT 06520 USA; 3 Image-Guided Therapy Systems, Philips Healthcare, Best, The Netherlands; 4 U/S Imaging and Interventions, Philips Research North America, Cambridge, MA USA

**Keywords:** Interventional oncology, Transarterial chemoembolization/embolisation (TACE), Radio-embolization/radio-embolisation, Liver/hepatic, Cancer, Imaging

## Abstract

**Purpose:**

To compare the visibility of liver metastases on dual-phase cone-beam CT (DP-CBCT) and digital subtraction angiography (DSA), with reference to preinterventional contrast-enhanced magnetic resonance imaging (CE-MRI) of the liver.

**Methods:**

This IRB-approved, retrospective study included 28 patients with neuroendocrine (NELM), colorectal (CRCLM), or sarcoma (SLM) liver metastases who underwent DP-CBCT during intra-arterial therapy (IAT) between 01/2010 and 10/2014. DP-CBCT was acquired after a single contrast agent injection in the tumor-feeding arteries at early and delayed arterial phases (EAP and DAP). The visibility of each lesion was graded by two radiologists in consensus on a three-rank scale (complete, partial, none) on DP-CBCT and DSA images using CE-MRI as reference.

**Results:**

47 NELM, 43 CRCLM, and 16 SLM were included. On DSA 85.1, 44.1, and 37.5 % of NELM, CRCLM, and SLM, were at least partially depicted, respectively. EAP-CBCT yielded significantly higher sensitivities of 88.3 and 87.5 % for CRCLM and SLM, respectively (*p* < 0.01), but not for NELM (89.4 %; *p* = 1.0). On DAP-CBCT all NELM, CRCLM, and SLM were visible (*p* < 0.001). Complete depiction was achieved on DSA for 59.6, 16.3, and 18.8 % of NELM, CRCLM, and SLM, respectively. The complete depiction rate on EAP-CBCT was significantly higher for CRCLM (46.5 %; *p* < 0.001), lower for NELM (40.4 %; *p* = 0.592), and similar for SLM (25 %, *p* = 0.399). On DAP-CBCT however, the highest rates of complete depiction were found—NELM (97.8 %; *p* = 0.008), CRCLM (95.3 %; *p* = 0.008), and SLM (100 %; *p* < 0.001).

**Conclusion:**

DAP-CBCT substantially improved the visibility of liver metastases during IAT. Future studies need to evaluate the clinical impact.

## Introduction

Cancer is a major health problem, nowadays being the most common cause of death of patients younger than 85 years in developed countries [1]. Metastatic liver disease is the most common cause of malignant liver lesions [2]. Independent of the primary tumor, many patients with metastatic liver disease are not eligible for liver resection [3, 4]. In addition, many of these patients present with chemoresistant disease such that the lesions show progression despite systemic chemotherapy. And in many of these patients, the oncologic disease is liver-dominant, where liver failure due to destruction of healthy liver tissue by the liver metastases is the primary limit to the patients’ life-expectancy. For these patients, intra-arterial therapies (IAT) such as transarterial chemoembolization (TACE) or radio-embolization (RE) are effective salvage therapies for inoperable liver metastases of different origin, such as colorectal cancer [5, 6], gastroenteropancreatic neuroendocrine tumors [7, 8], and sarcomas [9].

Contrast-enhanced magnetic resonance imaging (CE-MRI) is the imaging modality of choice for the diagnosis and therapy response assessment of primary and secondary liver cancer [10, 11]. Some liver metastases are hypervascular and show strong enhancement on CE-MRI, e.g., neuroendocrine liver metastases (NELM). However, most liver metastases present with a hypovascular, necrotic core and a viable, hypervascular rim, e.g., colorectal liver metastases (CRCLM). These hypovascular liver lesions are often occult or difficult to identify on digital subtraction angiography (DSA) images [12, 13], making the transition of preprocedure CE-MRI findings into the IAT often challenging, which might result in a less selective/precise IAT (e.g., lobar injection) with a higher chance of nontarget embolization and inadequate treatment.

Since the introduction of C-arm cone-beam computed tomography (CBCT) in Interventional Radiology [14, 15], this imaging modality has shown great value in the management of hepatocellular carcinoma (HCC) [16–20]. In particular, CBCT facilitates treatment planning and treatment delivery by providing a three-dimensional visualization of the tumor-feeding arteries and the capability to detect HCC lesions that are occult on DSA [12, 13]. IAT was initially developed for the treatment of HCC, so that most procedures are nowadays performed in patients with HCC. Thus, all studies that were investigating the detection capabilities of CBCT [19–23] were focusing on primary liver cancer and to our knowledge no study on the visibility of liver metastases on CBCT was published. However, liver metastases are often hypovascular and thus their visibility on DSA is not as conspicuous as compared to HCC. Thus, it is important to investigate and optimize the capabilities of CBCT for the intraprocedural visualization of liver metastases so that CBCT can facilitate IAT of liver metastases as well. Therefore, the purpose of our study was to compare the visibility of liver metastases on dual-phase cone-beam CT (DP-CBCT) and DSA with reference to preinterventional CE-MRI of the liver.

## Materials and Methods

### Study Cohort

This single-center, retrospective study was compliant with the Health Insurance Portability and Accountability Act and was approved by the Institutional Review Board. Informed consent was waived. All patients referred to IAT were discussed at our multidisciplinary liver tumor board. Between January 2010 and October 2014, a total of 1488 IATs were performed in 962 patients with primary or secondary liver cancer at our institution. Inclusion criteria for IAT were as follows: Eastern Cooperative Oncology Group (ECOG) performance status ≤2; Child-Pugh classification A or B; focal or multifocal hepatic malignancy; no severe ascites; albumin >2.5 g/dl; alanine aminotransferase and aspartate aminotransferase <5 times the upper normal limit; total serum bilirubin <3.0 mg/dl; serum creatinine <2.0 mg/dl; platelet count ≥50,000/mm^3^; international normalized ratio ≤1.5; and left ventricular ejection fraction ≥50 %.

The majority of the patients had primary liver cancer being either hepatocellular (*n* = 661) or cholangiocellular (*n* = 66) carcinoma. The most common liver metastases were from neuroendocrine cancer (NELM; *n* = 166), followed by colorectal cancer (CRCLM; *n* = 53) and sarcoma (SLM; *n* = 16). All patients with liver metastases that were referred to our department had liver-dominant disease and had shown progression of the liver metastases during systemic therapy, thus intra-arterial procedures were performed as salvage therapies.

In 515 out of 1488 IAT procedures, a DP-CBCT was acquired to facilitate the optimal placement of the treatment catheter, 98 of these DP-CBCTs were acquired during an IAT of secondary liver cancer. To avoid statistical bias due to repeated measurements in patients who received more than one IAT procedure, only the first IAT of each patient with secondary liver cancer was included, resulting in 50 DP-CBCTs for further analysis.

21 patients with more than ten lesions were excluded due to limited capabilities of two-dimensional DSA to distinguish individual lesions in such patients. Another patient was excluded because he showed severe disease progression between baseline MRI and IAT.

On the basis of these criteria, the final study population included 28 patients, who were treated by conventional TACE (cTACE; *n* = 13), radio-embolization (RE; *n* = 9), and drug-eluting beads TACE (DEB-TACE; *n* = 6), respectively. For cTACE procedures, a solution containing 100 mg of cisplatin, 50 mg of doxorubicin, and 10 mg of mitomycin C in a 1:1 mixture with Lipiodol (Guerbet, France) was injected, followed by the administration of 100- to 300-μm-diameter microspheres (Embospheres, Merit Medical, USA). For RE, a shunt scan was performed using 5–6 mCi of 99mTC-labeled macroaggregated albumin at least 1 week prior to the infusion of Y90 microspheres (TheraSpheres^®^, MDS Nordion, Ottawa, Canada). For DEB-TACE, LC Beads (2 mL; BTG, Surrey, England) with a diameter of 100–300 mm were loaded with 100 mg of doxorubicin hydrochloride (25 mg/mL) and mixed with an equal volume of nonionic contrast material. Up to 4 mL of drug-eluting beads was administered. Baseline characteristics are summarized in Table 1.

**Table 1:** Baseline characteristics of the study cohort (*n* = 28)

Characteristic	Value (%)
No. of patients	28 (100)
Sex	
Female	12 (42.9)
Male	16 (57.1)
Age*	
All patients	59 ± 12 years
Female	59 ± 14 years
Male	59 ± 9 years
Eastern cooperative oncology group performance status	
Grade 0	14 (50.0)
Grade 1	10 (35.7)
Grade 2	4 (14.3)
Origin of hepatic metastases	
Neuroendocrine cancer	15 (53.6)
Colorectal cancer	10 (35.7)
Sarcoma	3 (10.7)
Number of hepatic lesions	
1	3 (10.7)
2–4	11 (39.3)
5–10	14 (50.0)
Hepatic metastases location	
Right lobe	10 (35.7)
Left lobe	1 (3.6)
Bilobar	17 (60.7)
Extrahepatic metastases	
Lymph nodes	14 (50.0)
Lung	7 (25.0)
Bones	3 (10.7)

Except where indicated, data represents numbers of patients, and numbers in parentheses are percentages

* Data represented as mean ± standard deviation

### MR Imaging Technique

All study patients underwent baseline MRI within 2 months before IAT (median 20 days, range 0–61) using a 1.5-T MRI unit (Magnetom Avanto, Siemens Medical Solutions, Forchheim, Germany). A phased-array torso coil was used for signal reception. Our institutional liver protocol was performed including axial T2-weighted fast spin-echo images, axial single-shot breath-hold gradient-echo diffusion-weighted echo-planar images, and axial breath-hold unenhanced and contrast-enhanced (0.1 mmol/kg intravenous gadodiamide [Omniscan; Amersham, Princeton, NJ]) T1-weighted three-dimensional (3D) fat-suppressed spoiled gradient-echo images in the arterial, portal venous, and delayed phases (20, 70, and 180 s after intravenous contrast administration, respectively).

### Intraprocedural Imaging (DSA and C-Arm DP-CBCT)

All IAT procedures were performed by a single interventional radiologist (JFG) with 19 years of experience in hepatic interventions, using our standard institutional protocol [24]. Briefly, access was gained in the femoral artery using the Seldinger technique. The celiac axis was then cannulated using a 5-F Simmons-1 catheter (Cordis, Miami Lakes, FL, USA) through which a 2.8 F Renegade HI-FLO microcatheter (Boston Scientific, Marlborough, MA, USA) was coaxially advanced. Several angiographic steps were performed to define the hepatic arterial anatomy, to determine portal venous patency and tumor enhancement. Injection rates were adapted to the estimated blood vessel diameter (1–3 ml/s).

All procedures were performed using an angiographic system (Allura Xper FD20, Philips Healthcare, Best, The Netherlands) equipped with the XperCT module, enabling C-arm CBCT acquisition and volumetric image reconstruction (Feldkamp back projection) [25], and the DP-CBCT prototype feature, allowing the acquisition of two sequential CBCT scans (in an early and a delayed arterial phase (EAP and DAP)) using only one contrast injection [26, 27]. Contrast injections (Oxilan 300 mg I/ml; Guerbet, France) were performed with a power injector (Medrad, Indianola, PA, USA). All patients underwent C-arm DP-CBCT with the microcatheter placed into the hepatic artery branch that led to the tumor-feeding vessels and was in the same position as the last-acquired DSA, just prior to the delivery of the chemo-embolic (for TACE) or diagnostic (for shunt scan performed prior to RE) agents. In particular, the position of the microcatheter tip was lobar and segmental in 13 (46.4 %) and 15 (53.6 %) patients, respectively. The area of interest was positioned in the system isocenter prior to the DP-CBCT scan. The acquisition parameters were set to 120 kVp tube voltage and 50–325 mA tube current, the latter being modulated automatically during the acquisition. The two scans were triggered at 3 and 28 s after a single injection of 20 ml of undiluted contrast agent with a flow rate of 2 ml/s. The patients were instructed to be at end-expiration apnea during each of the CBCT scans with free breathing between the early and the delayed arterial phase scans. Oxygen was administered to patients during the procedure to minimize the discomfort of breath holding. With the motorized C-arm covering a 240° clockwise arc at a rotation speed of up to 55°/s, 312 projection images (60 frames/s) were acquired in 5.2 s. On completion of the acquisition, the two-dimensional projections were automatically transferred to the reconstruction computer, where they were reconstructed into 3D volumetric images with an isotropic resolution of 0.65 mm^3^, a field of view (FOV) of 250^2^ × 194 mm, and a matrix size of 384^2^ × 296.

### Image Analysis

Image analysis was performed using a free viewer software (Osirix, Pixmeo, Bernex, Switzerland) by two interventional radiologists, both with 4 years of experience (RES and RD), who did not participate in the IAT procedures. The observers were allowed to alter the window/level and zoom levels of the images to optimize perception. Streak artifacts caused by breathing, the intra-arterial catheter, and other medical devices (e.g., intravenous catheters) were assessed on DP-CBCT images using a three-point scale (none, localized, extensive). Extensive artifacts were considered to affect the diagnostic quality of the CBCT scan, whereas the presence of localized artifacts was deemed acceptable for diagnosis.

151 hepatic metastases were identified on preinterventional CE-MRI. 4 lesions were outside the FOV of the CBCT acquisitions and were excluded from the analysis. In addition, because the CBCTs were not acquired from the proper hepatic artery, but rather more selectively from within the liver vasculature, only lobar or segmental contrast attenuation of the liver parenchyma was seen. Thus, lesions that were entirely situated in liver segments, not opacified by the contrast medium injection during the CBCT acquisition were excluded. Of note, lesions that had a dual supply from both the left and right hepatic arteries were not excluded if the injected contrast medium reached the tumor from one of the feeding arteries. Following this approach, 41 lesions were excluded, leaving a total of 106 lesions for final analysis.

Each lesion was examined on the arterial and the portal venous phase of the preinterventional MRI and the lesion diameters were measured on the phase offering the best visualization of the lesion’s rim. Using this MRI phase as a side-by-side reference, the visibility of each lesion on DSA, EAP-, and DAP-CBCT was ranked according to the following scoring system: (1) optimal = the lesion was clearly detectable such as that in CE-MRI; (2) suboptimal = complete extent of the lesion was not visible; and (3) nondiagnostic = the lesion could not be detected at all.

### Statistical Analysis

All statistical computations were performed in SPSS Statistics 22 (IBM Corp., Armonk, NY). A *p* value less than 0.05 was considered statistically significant. Descriptive statistics were used to summarize the data. The distribution of all scale variables was assessed with the Shapiro–Wilk test. Scale variables with normal distribution were expressed with mean and standard deviation. For scale variables with nonGaussian distribution, median and range were reported. For ordinal variables, frequencies and percentage were used. Statistical significance was assessed with Friedman’s two-way ANOVA. In addition, binary testing of detected vs. not detected was performed using Cochran’s Q test after combining the categories for partial and complete depiction into one group.

## Results

On all CBCT images, localized streak artifacts were caused by contrast-filled catheters and contrast-enhanced arteries within the liver. The majority of EAP- and DAP-CBCT images showed no breathing artifacts (71 and 57 %, respectively), localized breathing artifacts were present in the remaining cases. In two patients (7 %), localized streak artifacts caused by the central venous catheter in the right atrium of the heart were observed. There were no extensive artifacts due to breathing, contrast-filled catheters, or other implants. Thus, all CBCT images were of diagnostic quality.

The median size of all metastatic liver lesions was 20 mm (range, 7–154 mm), with NELM lesions being slightly bigger (median 26 mm, range 10–116 mm) than SLM lesions (median 19 mm, range 8–100 mm) and CRCLM lesions (median 16 mm, range 7–154 mm). Out of the 106 lesions, only 65 (61.3 %) could be identified on DSA images, whereas EAP- and DAP-CBCT images depicted 94 (88.7 %) and 106 (100.0 %) lesions, respectively. Combining all metastatic liver lesions together, Cochran’s Q test showed that both EAP- and DAP-CBCT yielded significantly superior detectability compared to DSA (*p* < 0.01). Looking at each tumor entity separately, EAP-CBCT had only significant benefit for the detection of CRCLM and SLM, but not NELM, whereas DAP-CBCT was significantly better than DSA in detecting all the three metastases types (Table 2). However, DAP-CBCT had no significant advantage over EAP-CBCT for the detection of all lesions (*p* = 0.085), CRCLM (*p* = 0.689), NELM (*p* = 0.091), and SLM (*p* = 1.0).

**Table 2:** Detectability scores cross table of liver metastases on digital subtraction angiography (DSA), early arterial and delayed arterial phase (EAP- and DAP-) CBCT

Cancer type			EAP-CBCT					DAP-CBCT				
			1	2	3	Q	ANOVA	1	2	3	Q	ANOVA
Colorectal cancer	DSA	1	3	3	1	<0.001	0.008	7	0	0	<0.001	0.008
		2	5	6	1			11	1	0		
		3	12	9	3			23	1	0		
Neuroendocrine cancer	DSA	1	15	13	0	1.0	0.592	28	0	0	0.007	0.008
		2	3	8	1			12	0	0		
		3	1	2	4			6	1	0		
Sarcoma	DSA	1	2	0	1	0.009	0.399	3	0	0	0.001	<0.001
		2	0	3	0			3	0	0		
		3	2	7	1			10	0	0		

Detectability scores: 1 = complete depiction; 2 = partial depiction; 3 = no depiction

Q corresponds to Cochran’s Q test, performed after binary conversion of the scores (1 + 2=detected; 3 = not detected)

ANOVA corresponds to Friedman’s two-way ANOVA

More specifically, a complete depiction was achieved by DSA, EAP-, and DAP-CBCT in 38 (35.8 %), 43 (40.6 %), and 103 (97.2 %) liver metastases, respectively. Partial depiction was achieved on DSA, EAP-, and DAP-CBCT images for 27 (25.5 %), 51 (48.1 %), and 3 (2.8 %) liver metastases, respectively. Friedman’s Two-Way ANOVA showed a significant advantage of DAP-CBCT over EAP-CBCT (*p* < 0.001) and DSA (*p* < 0.001), respectively, whereas the difference between EAP-CBCT and DSA was not significant (*p* = 0.298). Looking at each tumor entity separately, EAP-CBCT significantly outperformed DSA only for the complete depiction of CRCLM, but not of NELM and SLM (Table 2). DAP-CBCT on the other hand, was not only significantly better than DSA for the complete depiction of all the three types of metastatic liver lesion, but also better than EAP-CBCT for the complete delineation of CRCLM (*p* = 0.008), NELM (*p* < 0.001), and SLM (*p* = 0.031).

**Fig. 1:**
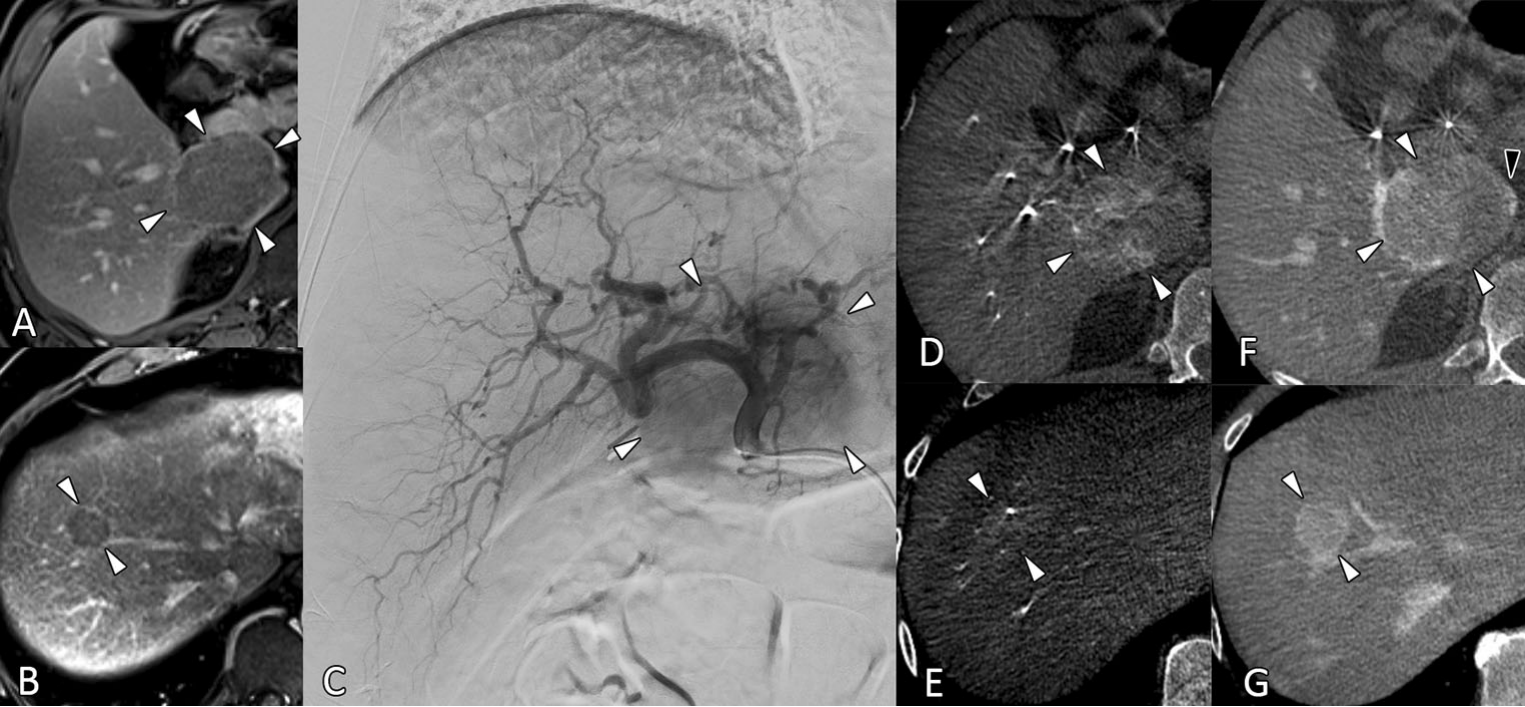
54-year-old man with a history of neuroendocrine cancer of the small bowel with liver metastases, treated using conventional TACE. Contrast-enhanced T1-weighted gradient-echo sequence in the portal venous phase shows a large, rim-enhancing lesion in the caudate lobe (**A**, *arrowheads*) and a smaller lesion of similar pattern in segment 8 (**B**, *arrowheads*). On DSA images acquired with the microcatheter tip in the proper hepatic artery, only the large lesion could be identified (**C**, *arrowheads*). On early arterial phase CBCT images, only the lateral parts of the large lesion are depicted (**D**, *arrowheads*), the smaller lesion is only silhouetted against the surrounding parenchyma (**E**, *arrowheads*). On delayed arterial phase CBCT images, the large lesion is well depicted (**F**) to include both the lateral parts (*white arrowheads*) as well as the medial rim (*black arrowhead*). Of note, the small lesion is completely depicted (**G**, *arrowheads*)

**Fig. 2:**
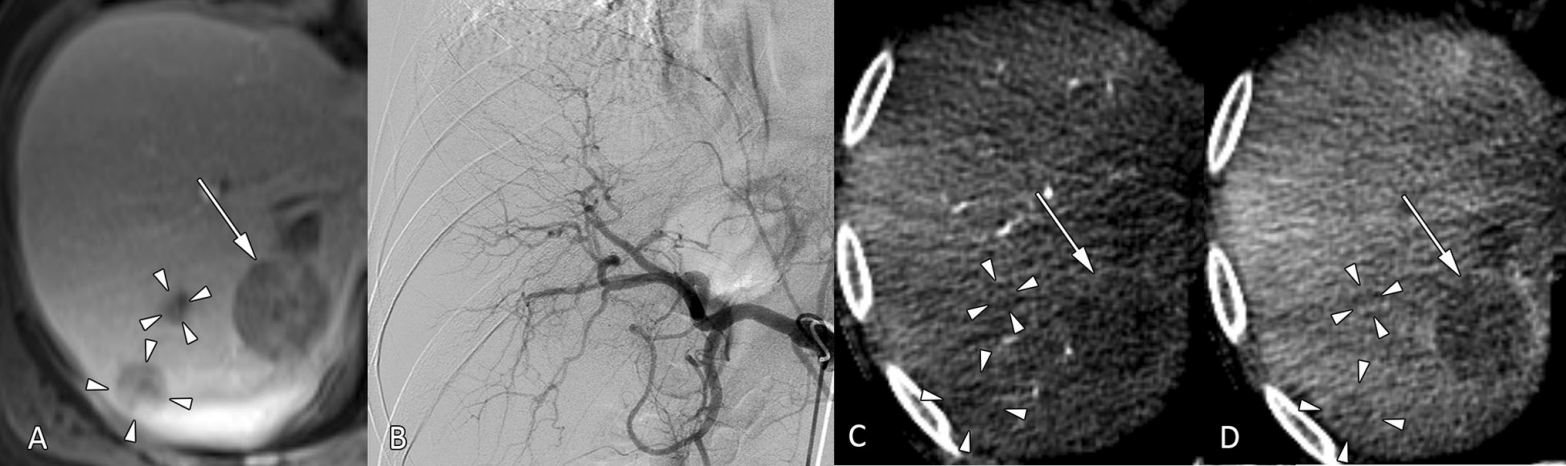
43-year-old man with a history of retroperitoneal sarcoma with liver metastases, treated using conventional TACE. **A** Contrast-enhanced T1-weighted gradient-echo sequence in the portal venous phase shows three lesions in segment 7, one larger (*arrow*) and two smaller tumors (*arrowheads*). **B** On the celiac arteriogram, none of the lesions is visible. **C** On early arterial phase CBCT, the large lesion is well depicted (*arrow*), but the two smaller lesions are difficult to distinguish (*arrowheads*). **D** On delayed arterial phase CBCT, all three lesions are well depicted (*arrow* and *arrowheads*)

**Fig. 3:**
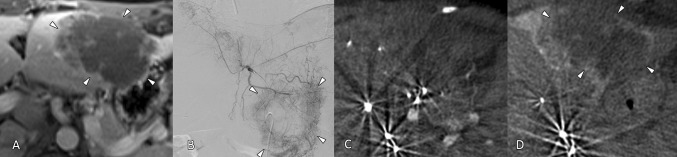
44-year-old woman with a history of colorectal cancer with liver metastases, treated using radio-embolization with Yttrium-90. **A** Contrast-enhanced T1-weighted gradient-echo sequence in the portal venous phase shows a large, mainly necrotic lesion with rim enhancement in segment 3. **B** On the DSA images acquired with the microcatheter tip in the left hepatic artery, the lesion is well depicted. **C** However, on early arterial phase CBCT images, the lesion is not visible. **D** On delayed arterial phase CBCT images, the entire extent of the lesion is well depicted (*arrowheads*)

All 41 lesions missed by DSA were detected by DAP-CBCT, 39 (95 %) and 2 (5 %) being completely and partially depicted, respectively (Fig. 1). Out of these 41 lesions, 15 (36.6 %) and 18 (43.9 %) were completely and partially depicted on EAP-CBCT, respectively (Fig. 2). DAP-CBCT depicted 12 more lesions than EAP-CBCT, whereas EAP-CBCT did not show any additional lesions compared to DAP-CBCT. All four lesions, that were missed on EAP-CBCT, but visible on DSA, were completely depicted on DAP-CBCT (Fig. 3).

## Discussion

The main finding of our study was that DAP-CBCT substantially improved the detectability of all three entities of metastatic liver lesions during IAT procedures. Using conventional DSA alone, almost 40 % of the liver metastases could not be identified. Whereas EAP-CBCT missed only approximately 10 % of the liver metastases and DAP-CBCT depicted all of them. Most likely, without the additional information provided by the CBCT scans, a less selective treatment (e.g., lobar application) would have been performed in some of the patients. This brings the disadvantage of exposing more healthy tissues (nontargeted treatment) to the drug payload and having a greater chance of undertreatment.

Previous publications that compared standard CBCT (using only one arterial phase) to conventional computed tomography showed that 89 % of HCC lesions and especially the majority of small HCC lesions that were invisible on conventional DSA could be identified [19, 28]. With the addition of DAP-CBCT, the detection rate of HCC lesions increased slightly to 93.9 %, in comparison to CE-MRI [22]. Another study investigated the detectability of intrahepatic cholangiocarcinoma (ICC) lesions on DP-CBCT, using CE-MRI as Ref. [23]. This study showed that due to delayed enhancement pattern of ICC lesions, as seen on DSA and MRI [29, 30], DAP-CBCT was substantially better in depicting ICC lesions than EAP-CBCT and DSA. Similar to ICC, most metastatic liver lesions are rather hypovascular and show delayed enhancement pattern which is often limited to a rim around a necrotic core, whereas NELM are often hypervascular lesions [10, 11]. Thus, less than 50 % of CRCLM and SLM lesions, but 85 % of NELM could be identified on DSA in our study. On CBCT, most lesions showed minimal to no enhancement on EAP-CBCT, with the lesion rim and the surrounding liver parenchyma enhancing on DAP-CBCT, demarcating the necrotic core of these lesions.

Although the detection rate for NELM was already quite high on DSA, DAP-CBCT reached a significantly higher detection rate for all the three entities of metastatic liver lesions, whereas EAP-CBCT outperformed DSA only for CRCLM and SLM, but not for NELM lesions.

Recent publications have shown that intraprocedural CBCT does not only facilitate the positioning of the delivery catheter for optimal targeting of the tumor [31], but also provides intraprocedural feedback on the technical success of the IAT procedure by means of three-dimensional quantification of contrast enhancement/deposition [32, 33]. For that purpose, a partial depiction of intrahepatic lesions is not sufficient, only lesions with a complete depiction can be evaluated using this new approach. In our study, EAP-CBCT delineated only 40 % of the lesions completely, whereas DAP-CBCT succeeded in 97 %. This underlines the importance of an optimized CBCT acquisition protocol, based on tumor enhancement patterns.

Additional radiation exposure is often considered a severe drawback of CBCT. However, a recently published trial that investigated the radiation exposure during TACE showed that CBCT accounts for only approximately 10 % of the radiation exposure during the entire procedure using standard equipment [34]. In particular, a single-phase CBCT corresponds to approximately 150 s of digital fluoroscopy or 4 s of DSA. A CBCT run with the catheter tip in the proper hepatic artery could be used as the source of a three-dimensional overlay for intraprocedual guidance and could in theory replace all intrahepatic DSA runs, thereby reducing both radiation exposure and contrast volume. However, this needs to be confirmed in a prospective trial.

The present study has some limitations: First, being the small sample size with metastases of different origins. However, the number of patients with secondary liver cancer being treated by means of IAT is rather small compared to the patient population with hepatocellular carcinoma. In addition, 44 % of the patients had to be excluded due to extensive disease, limiting the diagnostic capabilities of DSA. Although a less selective approach for IAT might be indicated in these patients, CBCT should still be performed to verify if known lesions have progressed or new liver lesions have emerged because this could modify the treatment plan to either a less selective drug delivery or to have additional selective catheter positions for drug delivery. Second, in the absence of a control group of patients with liver metastases treated using IAT without CBCT, the impact of CBCT on radiological response or overall outcome could not be evaluated within this retrospective study. However, the evaluation of the diagnostic accuracy of a new intraprocedural imaging modality is a prerequisite before prospective trials are conducted to assess the clinical impact. Third, the CBCTs were not acquired from the proper hepatic artery, but rather more selectively from within the liver vasculature, thus only lobar or segmental contrast attenuation of the liver parenchyma was seen and lesions in other segments as depicted by the CE-MRI had to be excluded. This also did not allow for investigation of the intraprocedural guidance capabilities of CBCT. However, the position of the catheter was selected based on the tumor burden as seen on the preinterventional CE-MRI. Fourth, some hepatic lesions had to be excluded because they were outside the FOV of the CBCT scan. This has been a common problem of CBCT until recently, with up to 12 % of lesions being outside the FOV in the literature [23, 28]. However, a solution to this problem was recently demonstrated by changing the CBCT rotation trajectory while still maintaining the same number of projection images and rotational sweep angle [35]. Fifth, for each patient, only one DP-CBCT was acquired prior to the delivery of embolic agents, no DP-CBCT was acquired after the delivery. Thus, quantitative intraprocedural response assessment as described previously [32, 33] could not be performed.

Despite these limitations, our results demonstrated that the addition of a second CBCT phase significantly improved identification of metastatic liver lesions. Although EAP-CBCT did not show any lesions missed by DAP-CBCT, the former is still necessary to visualize the feeding arteries in our current protocol [36]. However, an optimized DAP-CBCT protocol is currently underdevelopment, that uses a prolonged contrast injection to facilitate the visualization of both the feeding arteries and the tumor parenchyma [37].

In conclusion, DAP-CBCT substantially improved the visibility of liver metastases during IAT. Future studies need to investigate whether this improved visibility facilitates a more selective treatment, resulting in a better radiological response and better overall outcome. In addition, the capabilities of CBCT for intraprocedural guidance should be evaluated, thereby having the potential to replace intrahepatic DSA runs in order to reduce radiation exposure and allow for the assessment of treatment success during IAT of liver metastases.
